# Supplementary material for: Identification of atrial fibrillation associated genes and functional non-coding variants
Source: Nat Commun. 2019 Oct 18;10:4755. doi: 10.1038/s41467-019-12721-5 (PMC6802215; doi:10.1038/s41467-019-12721-5)
Supplement: Supplementary file 2 — Supplementary Information [file 41467_2019_12721_MOESM2_ESM.pdf]

Identification of atrial fibrillation associated genes and functional non-coding variants

Van Ouwerkerk et al.

Supplementary Information

Supplementary Figure 1

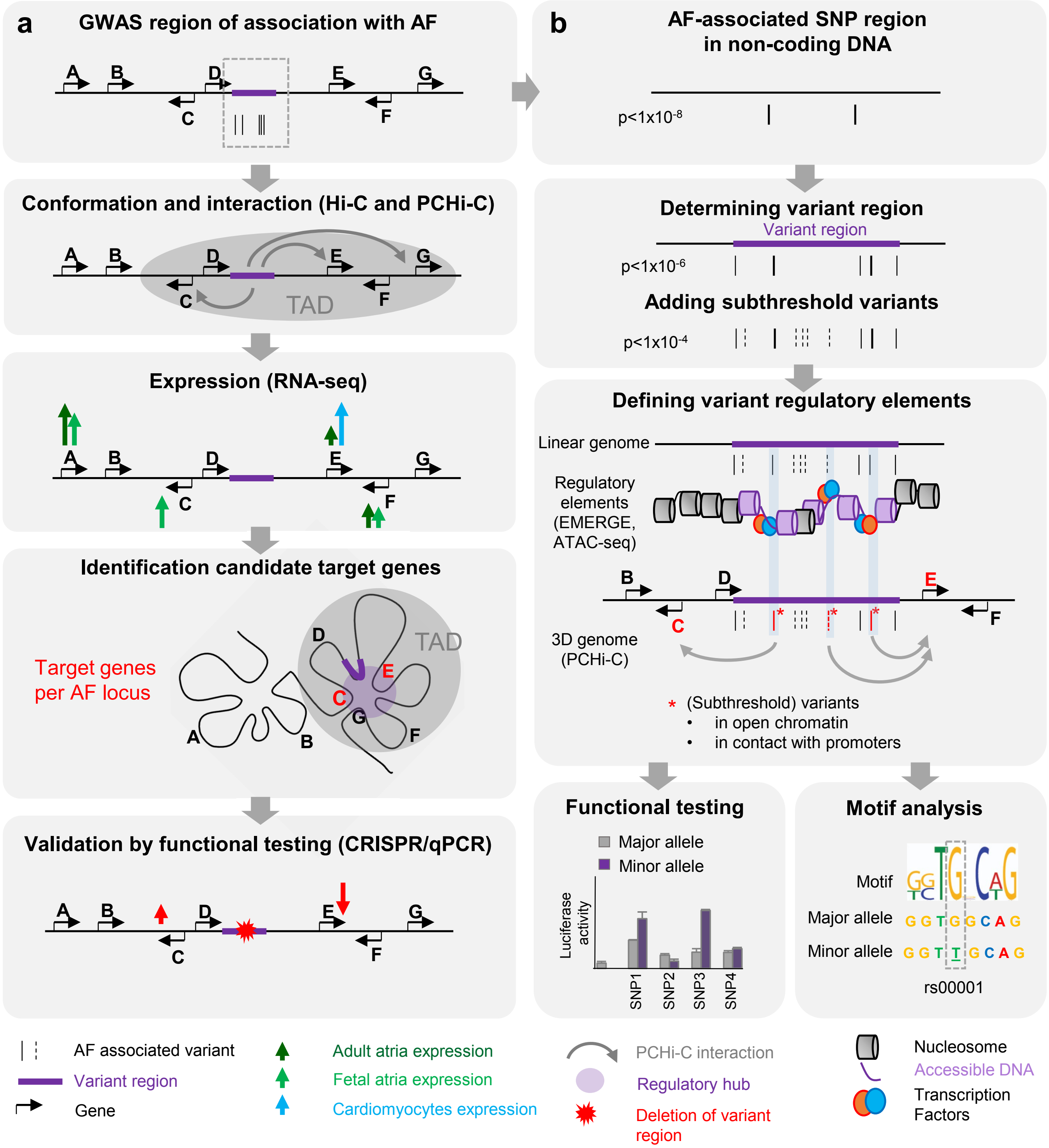

Supplementary Figure 1. Flowchart approach

a) GWAS to target genes. GWAS regions were plotted (top panel) to determine which genes lie within the locus TAD using Hi-C and PCHi-C, and expression of these genes was determined using several RNA-seq datasets. Combining this data, we determined likely candidate genes per AF locus. Furthermore, we validated the candidate genes by *in vivo* deletion of the LD block using CRISPR. **b)** GWAS to functional variants. Variant regions (VRs) were defined as first to last genome-wide significant variant per region. Subthreshold variants in the VRs were included to identify functional variants that lie in regions with epigenetics marks (EMERGE) or accessible DNA (ATAC-seq). Target genes of functional variants were identified using PCHi-C. Moreover, on the functional variants we performed motif analysis to identify variants that allele-specifically alter TF-binding sites. The approach was validated by functional testing. Abbreviations: Genome Wide Association Study (GWAS), Linkage Disequilibrium (LD), Promoter Capture Hi-C (PCHi-C), Atrial Fibrillation (AF), Topologically Associated Domain (TAD), Transcription Factor (TF).

Supplementary Figure 2

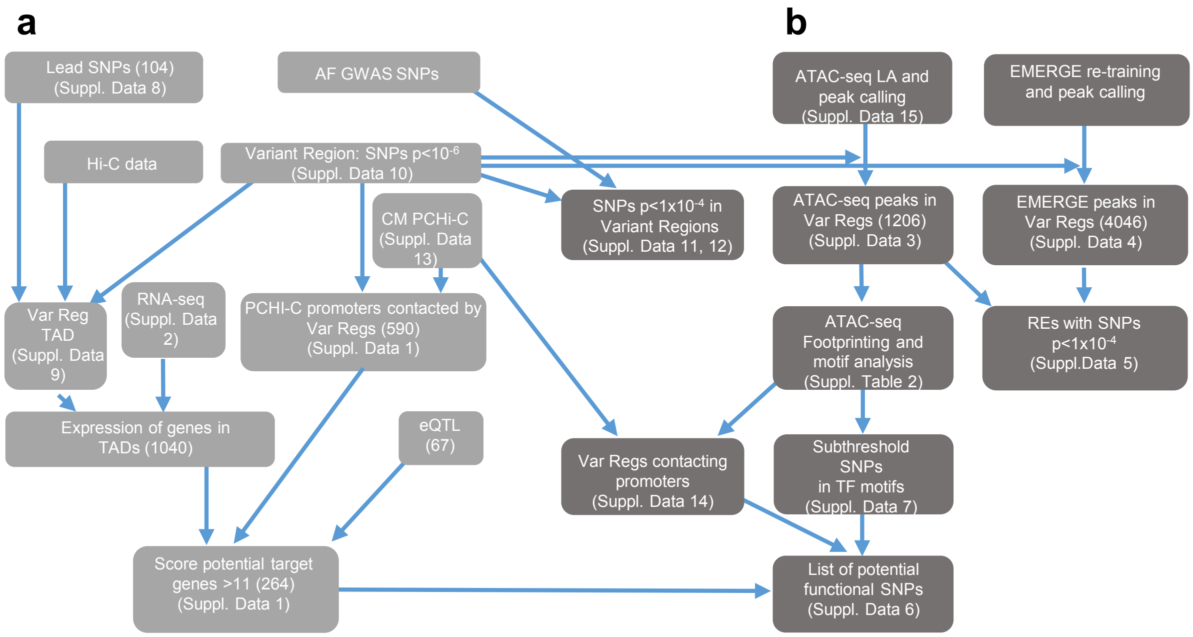

Supplementary Figure 2. Flowchart bioinformatics approach

Flowchart of bioinformatics approach showing datasets used, reference to supplementary tables and data (results in brackets) of **a)** target gene identification, and **b)** variant regulatory element identification.

Supplementary Fig. 3. Variant regions as determined by different p-value cutoffs and LD analysis

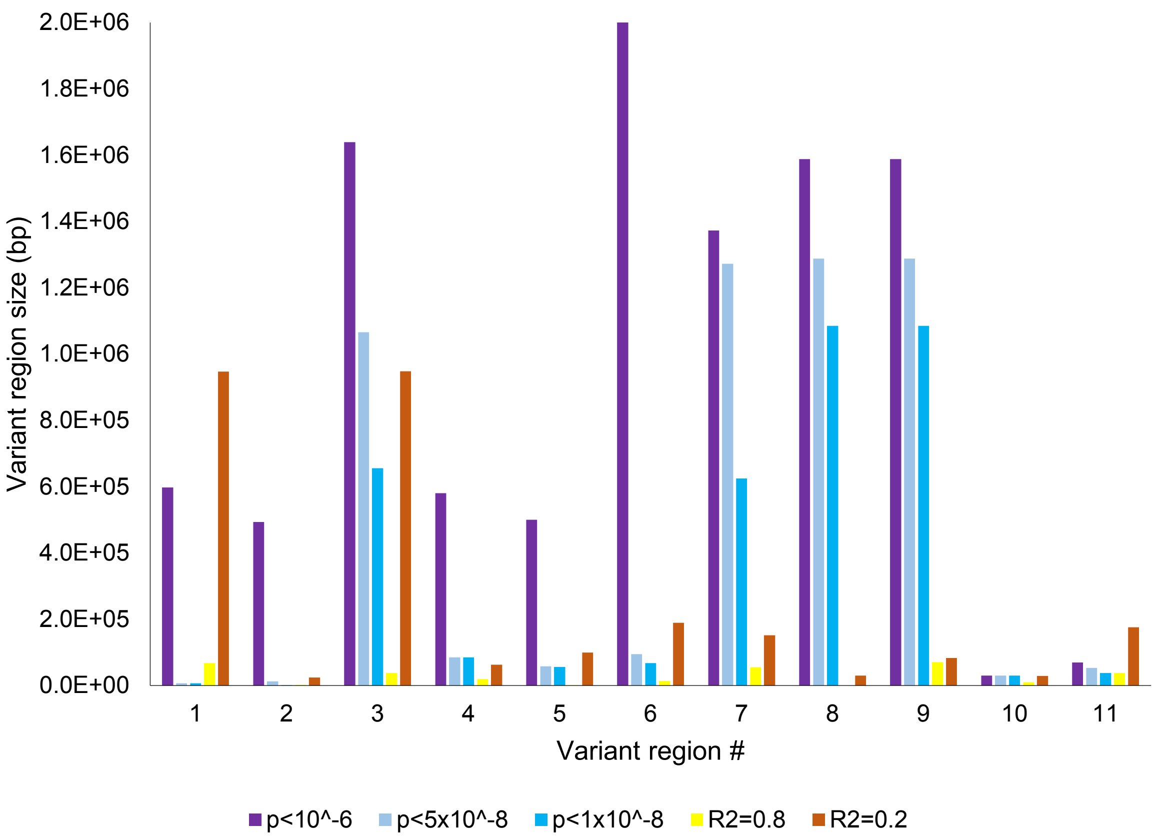

Supplementary Figure 3. Variant regions as determined by different p-value cutoffs and LD analysis

Variant region sizes as determined by GWAS p-value cut-offs as well as stringent ($R2=0.8$) and lenient ($R2=0.8$) LD around the sentinel SNP.

Supplementary Fig. 4. Normalized expression and PCHi-C data

a

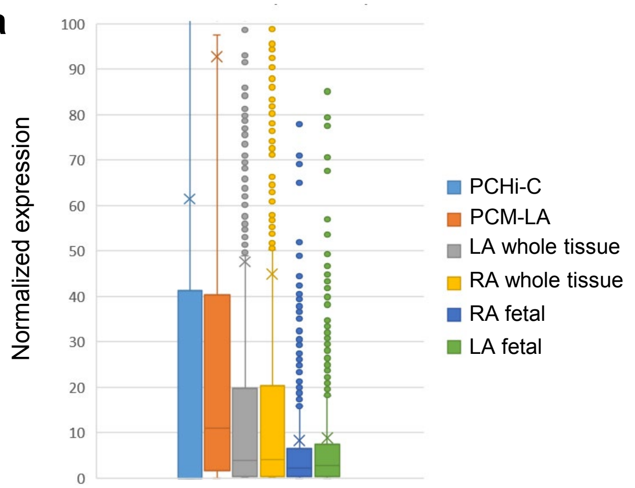

b

Dataset	No Expression (0)	Expression (1)	High expression (2)
PCHi-C	0<>0 tags	>0 tags	-
LA-PCM	0<>10 tags	10<>40 tags	>40 tags
LA whole tissue adult	0<>4 tags	4<>20 tags	>20 tags
RA whole tissue adult	0<>3 tags	3<>20 tags	>20 tags
RA fetal	0<>3 tags	3<>8 tags	>8 tags
LA fetal	0<>3 tags	3<>8 tags	>8 tags

Supplementary Figure 4. Normalized expression and PCHi-C data

a) Boxplot showing normalized counts and spread of counts per genome-wide dataset (five RNA-seq, one PCHi-C). **b)** Boundaries per expression category used for the scoring of potential target genes.

Supplementary Fig. 5

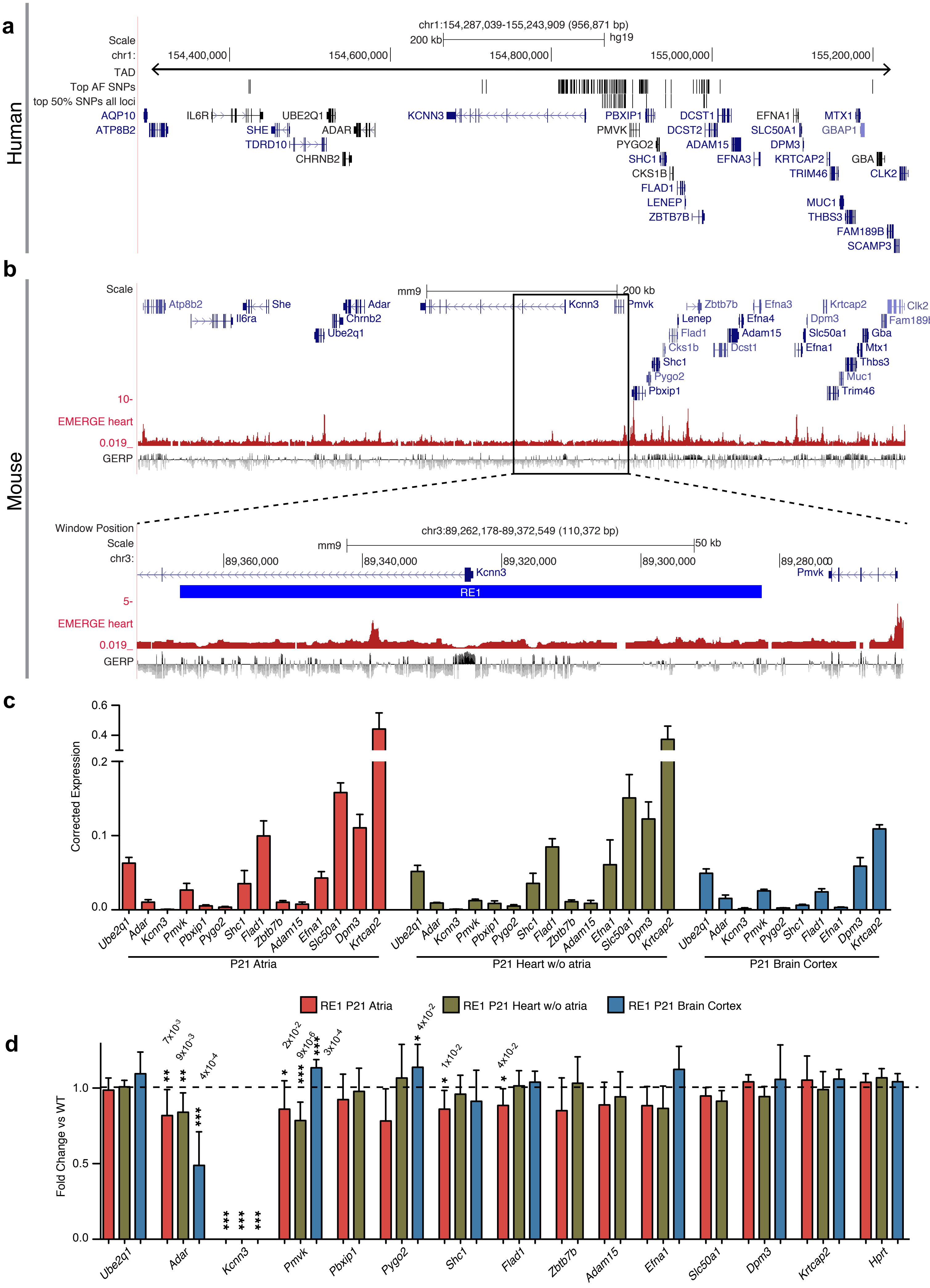

Supplementary Figure 5. Absence of an AF variant region in the 1q21 locus (*Kcnn3*) leads decreased expression within the TAD.

a) Human 1q21 locus (*KCNN3*) showing top SNPs. **b)** Homologous region in the mouse (3qF1) with EMERGE enhancer prediction track (top) and zoom in to show the 85Mb deletion RE1 with conservation track. **c)** Wild-type expression of candidate affected genes in the TAD in P21 left and right atria and heart without atria. N=8 wildtype, n=9 homozygotes for all tissues. Source data are provided as Source Data file. **d)** Expression fold change of $Kcnn3^{RE1/RE1}$ vs wild-type littermates with *Hprt* as a negative control. Error bars represent SD, * is p<0.05, ** is p<0.01, and *** is p<0.001. Source data are provided as Source Data file.

Supplementary Fig. 6

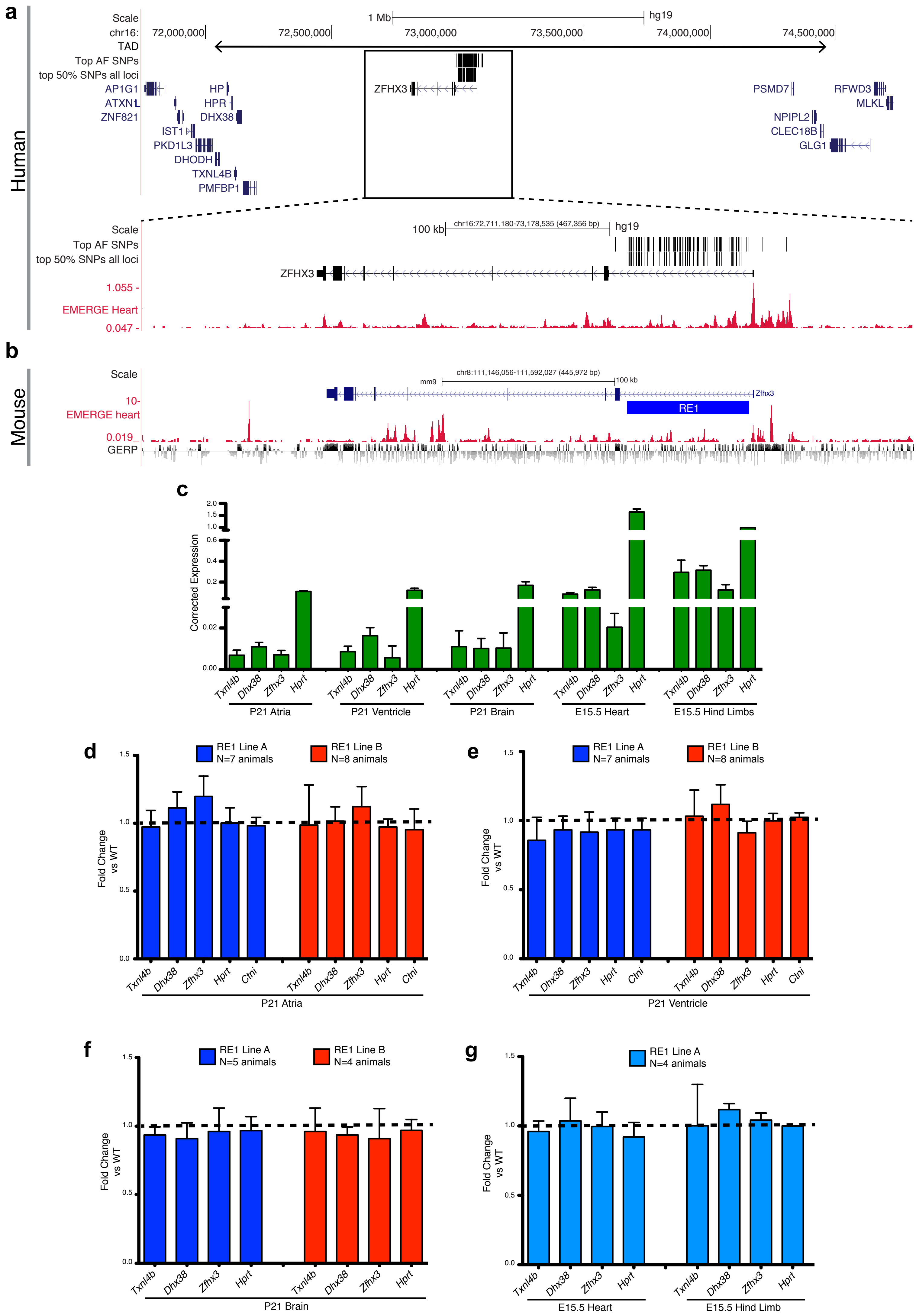

Supplementary Figure 6. 33Mb region deletion in the first intron of *Zfhx3* has no effect in candidate gene expression.

a) Human 16q22 locus showing SNPs and EMERGE track. **b)** Homologous region in the mouse showing the 33Mb deletion in the first intron on *Zfhx3*. **c)** Wild-type corrected expression of candidate genes in the TAD of P21 left and right atria, ventricles, and brain, and E15.5 whole hearts and hind limbs. **d)** Expression fold change of two independent $Zfhx3^{RE1/RE1}$ mouse lines vs wild-type littermates of P21 atria, **e)** P21 ventricles, **f)** P21 brain, and **g)** E15.5 whole hearts and hind limbs of line A only. Error bars represent standard deviation (SD), p-values determined using two-tailed Student's t-test. Source data of panels c-g are provided as Source Data files.

Supplementary Fig. 7

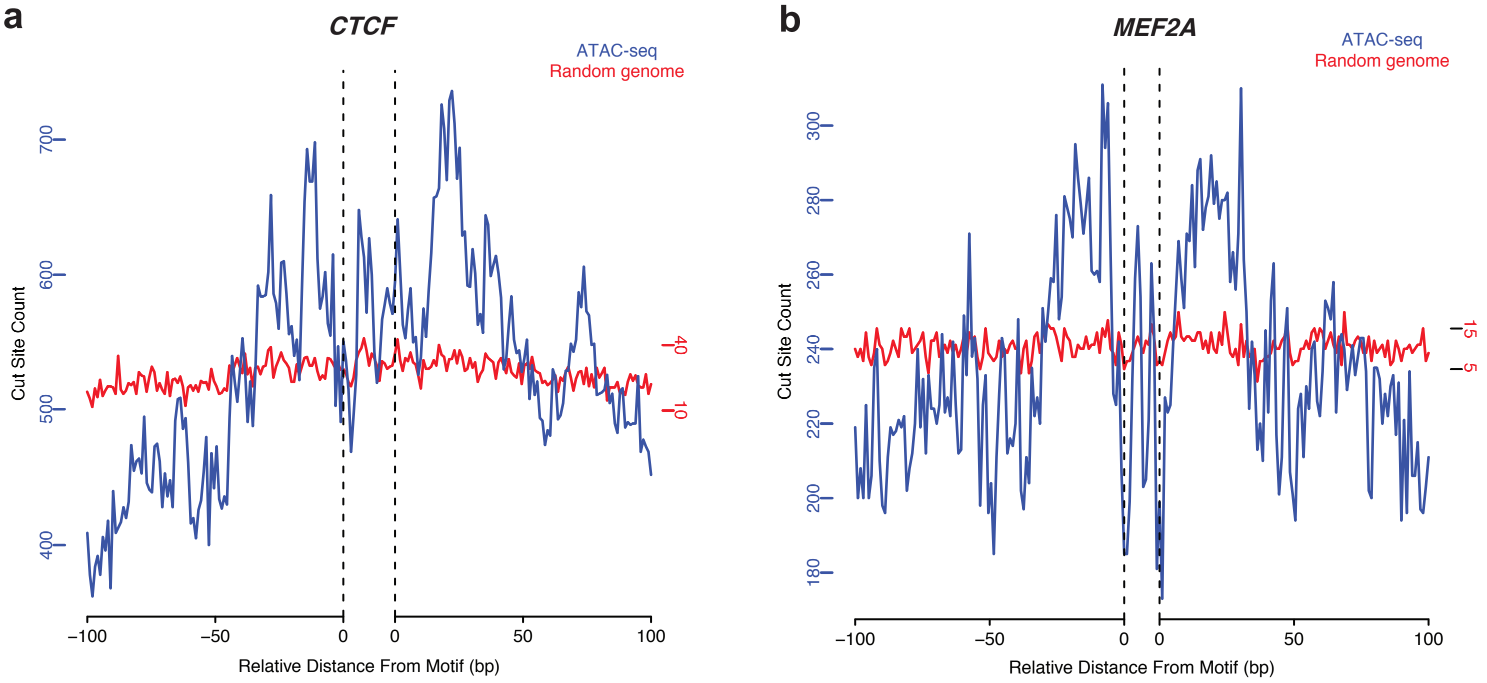

Supplementary Figure 7. ATAC-seq footprinting of *CTCF* and *MEF2A*.
a) The footprint of *CTCF* and **b**) *MEF2A* in ATAC-seq compared to random genome.

Supplementary Fig. 8

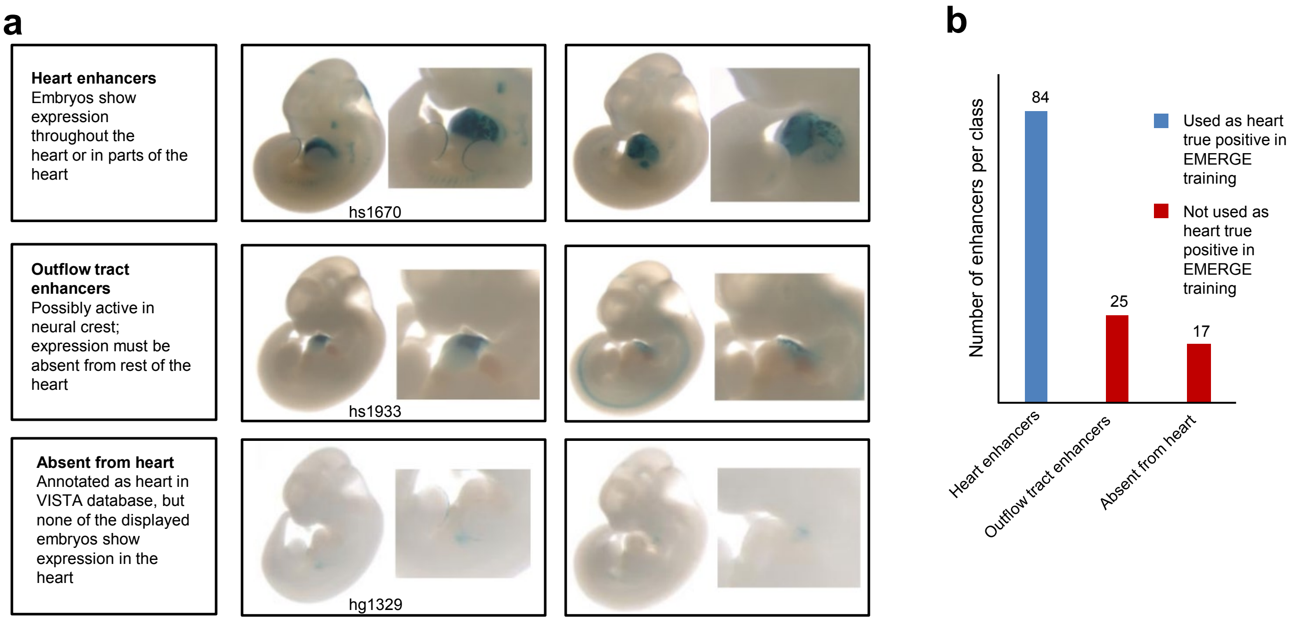

Supplementary Figure 8. Curation of validated heart enhancers prior to heart enhancer prediction.
a) The annotation criteria per activity class are given, as well as a representative example of each class. The image of the enhancer screened transgenic embryos are taken from the Vista enhancer browser [73]. **b**) The number of enhancers that were found per class and which class was used as true positive training data for subsequent heart enhancer prediction.

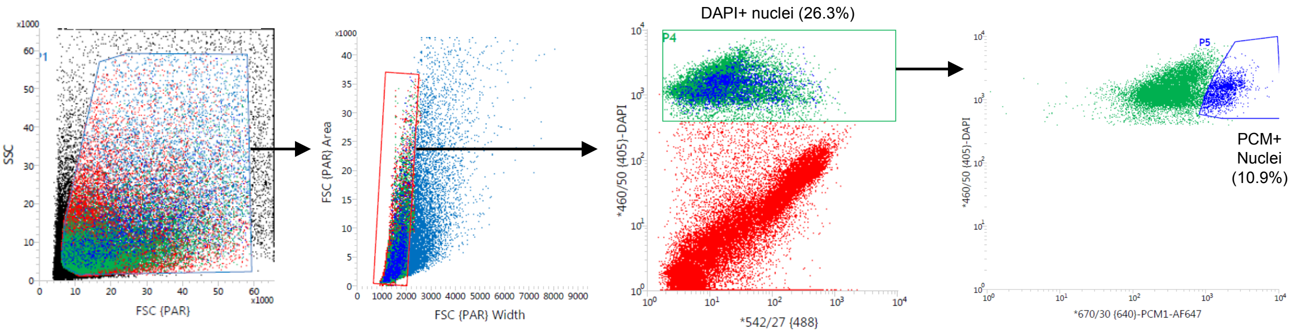

Supplementary Figure 9. FACS gating strategy for sorting PCM1⁺/DAPI⁺ nuclei of left atria
Gating strategy to isolate intact viable nuclei that are DAPI and PCM1 positive for RNA-seq presented in Fig. 1d,e, Fig. 3a,e, Fig. 4b and Fig. 5d.

Supplementary Table 1: Summary of (epi)genetic data per AF-associated locus

VR#	sentinel SNP	chr	length variant region ($p<10^{-6}$) (bp)	length variant region ($p<5x10^{-8}$) (bp)	length variant region ($p<1x10^{-8}$) (bp)	TAD length (bp)	genes in TAD	genes contacted by variant region	ATAC-seq peaks	EMERGE peaks
1	rs187585530	chr1	6160	6160	6160	597299	13	2	1	1
2	rs880315	chr1	11951	11951	319	493010	13	2	2	7
3	rs146518726	chr1	1177831	1065803	655450	1639190	9	9	12	25
4	rs1443926	chr1	92003	84978	84978	580500	1	0	12	4
5	rs4484922	chr1	76421	57803	56130	499875	4	2	8	12
6	rs79187193	chr1	161041	94584	67261	3632299	10	4	14	19
7	rs11264280	chr1	701723	1272754	624890	878812	28	37	39	62
8	rs72700114	chr1	1086897	1287590	1084665	1587911	12	6	32	40
9	rs503706	chr1	1086897	1287590	1084665	1587911	14	6	32	40
10	rs4590732	chr1	30192	30192	30192	233813	7	11	2	14
11	rs4951261	chr1	68897	52589	37606	1034665	11	6	3	6
12	rs7919685	chr10	500205	525326	496009	862687	4	3	9	15
13	rs6480708	chr10	689834	292503	267052	889010	23	15	29	42
14	rs11001667	chr10	73303	54109	54109	1826157	6	1	1	1
15	rs1044258	chr10	726740	434201	246797	1124992	28	36	32	40
16	rs11598047	chr10	713418	471102	471102	1571804	29	35	47	100
17	rs1822273	chr11	37510	31775	31775	197531	1	1	4	6
18	rs949078	chr11	88494	88494	88494	915093	4	0	2	2
19	rs76097649	chr11	32687	32687	30183	1338375	8	1	6	8
20	rs10842383	chr12	204179	152642	120980	2172009	3	0	3	1
21	rs113819537	chr12	54940	2903	2903	661125	4	0	3	2
22	rs12809354	chr12	86012	86012	86012	11639132	6	2	8	24
23	rs7978685	chr12	188091	186910	175444	709499	19	22	9	12
24	rs35349325	chr12	243219	230810	230798	1632656	13	10	16	17
25	rs11180703	chr12	40744	40744	33724	1386750	6	1	1	1
26	rs883079	chr12	408514	388254	103957	600657	4	2	21	13
27	rs12810346	chr12	463943	290723	17119	1435125	4	2	6	9
28	rs12298484	chr12	96070	95773	78487	580977	4	9	7	12
29	rs9580438	chr13	688391	63560	53223	1793906	1	1	4	6
30	rs28631169	chr14	73087	71970	71970	431343	16	13	5	14
31	rs2145587	chr14	101981	101981	101981	1136813	3	1	5	12
32	rs73241997	chr14	52814	52814	40018	1281938	11	2	2	2
33	rs2738413	chr14	265420	263700	263700	1644751	16	8	11	18
34	rs10873299	chr14	9021	4604	4604	612750	11	1	1	5
35	rs62011291	chr15	88100	4494	4494	1056187	14	6	1	2
36	rs12591736	chr15	4437	2019	0	1394813	6	0	1	1
37	rs7172038	chr15	309356	211154	211154	1277907	13	3	9	29
38	rs12908004	chr15	47552	11273	11273	693374	5	0	1	0
39	rs12908437	chr15	48636	46621	41685	644716	3	1	5	16
40	rs2286466	chr16	137956	262255	117985	987553	52	34	19	45
41	rs2359171	chr16	649635	223416	223416	2350218	8	3	21	28
42	rs8073937	chr17	311307	108367	105819	890905	61	46	25	55
43	rs72811294	chr17	300000	0	0	1060218	6	2	14	25
44	rs242557	chr17	1035395	1035395	0	1433098	15	11	29	14
45	rs7219869	chr17	143338	14073	10730	1092468	2	1	2	0
46	rs9953366	chr18	41724	13806	7878	1064249	4	1	4	6
47	rs6546620	chr2	67055	55425	34270	1100530	15	0	3	3
48	rs6742276	chr2	520267	480006	475628	1197281	14	6	10	9
49	rs2723064	chr2	173036	151129	151129	366844	4	2	8	11
50	rs6546553	chr2	619648	525038	383381	999750	18	12	20	33
51	rs72926475	chr2	398660	241293	198711	1991271	15	9	20	19
52	rs12992412	chr2	373109	41699	0	2036598	5	0	2	2
53	rs56181519	chr2	156580	156580	156580	354750	4	2	1	2
54	rs2288327	chr2	429119	348655	348655	2245406	12	6	64	94
55	rs295114	chr2	538568	419674	393818	939281	9	5	13	8
56	rs2145274	chr20	178987	34441	0	1797938	2	0	1	5
57	rs7269123	chr20	35025	0	0	793912	19	7	3	12
58	rs2834618	chr21	74805	74805	74805	1475436	5	0	1	0
59	rs465276	chr22	319686	314743	4011	1382717	18	9	52	88
60	rs7650482	chr3	280900	247735	247735	499876	8	7	28	36
61	rs73032363	chr3	18059	11118	0	2566666	7	0	1	0
62	rs6790396	chr3	722403	722403	722403	1048125	15	17	39	53
63	rs2306272	chr3	170743	54209	54209	711872	2	0	15	31
64	rs17490701	chr3	283834	184635	179731	399093	6	5	13	19
65	rs4855075	chr3	5507	5507	3475	653063	9	5	2	2
66	rs9872035	chr3	30682	0	0	638802	14	3	1	1
67	rs3822259	chr4	443541	24035	4488	1700498	4	1	13	33
68	rs3960788	chr4	399993	323098	283415	907032	9	5	12	11
69	rs6847935	chr4	1219388	1049429	879082	2139419	5	3	15	13
70	rs55754224	chr4	139401	115618	0	2088187	3	1	8	10
71	rs10213171	chr4	610766	137368	122034	2313937	4	1	26	30
72	rs10520260	chr4	254716	176480	176480	846562	4	4	16	16
73	rs716845	chr5	722017	36726	36726	1705219	5	1	6	5
74	rs10479177	chr5	883050	1075334	852454	977406	22	15	39	39
75	rs174048	chr5	356532	355429	355429	1261498	3	0	6	3
76	rs6882776	chr5	189934	150901	150901	645000	10	7	12	11
77	rs73366713	chr6	36748	36748	36748	855856	5	3	1	9
78	rs34969716	chr6	202360	202360	202360	1514842	11	3	4	3
79	rs1307274	chr6	1103	0	0	366843	4	1	0	0
80	rs3176326	chr6	14344	1701	1701	1285968	24	7	2	5
81	rs6907805	chr6	591697	186856	0	1862438	12	6	12	11
82	rs210632	chr6	350492	0	0	1241625	12	6	5	7
83	rs9481842	chr6	468297	491790	468297	1068117	7	5	18	25
84	rs868155	chr6	607691	784363	322087	1682220	5	1	7	10
85	rs12208899	chr6	802067	0	0	1108593	7	5	14	9
86	rs117984853	chr6	122340	91328	88571	1103041	7	4	2	5
87	rs11768850	chr7	43739	28530	0	900400	7	2	7	12
88	rs55734480	chr7	14523	13777	1705	1842281	2	1	0	0
89	rs6462078	chr7	28625	8001	8001	870748	2	0	1	3
90	rs74910854	chr7	138446	24206	0	1210672	7	0	5	1
91	rs62483627	chr7	483589	439762	20592	1003781	13	8	15	22
92	rs11773845	chr7	445841	440072	440072	761907	6	5	21	16
93	rs55985730	chr7	300000	0	0	1076132	20	14	12	36
94	rs7789146	chr7	106668	55042	49275	1479465	32	12	8	15
95	rs7508	chr8	251288	234737	223375	1592345	11	3	9	9
96	rs7846485	chr8	139915	110369	110369	2039607	18	9	5	5
97	rs62521286	chr8	449251	282391	282391	628875	13	11	25	31
98	rs35006907	chr8	25428	10475	8017	612751	7	11	3	6
99	rs7460121	chr8	43740	22229	0	427313	4	0	3	3
100	rs6993266	chr8	365331	344988	342369	834307	8	5	9	15
101	rs4977397	chr9	48926	23458	19299	1737469	9	0	0	1
102	rs7026071	chr9	710388	710388	710388	1241249	13	4	41	70
103	rs4743034	chr9	85939	11527	7617	1576219	2	0	5	7
104	rs10760361	chr9	39415	30422	0	640968	7	0	2	2
SUM			30990268	23247497	17169975	133506072	1040	618	1206	1750
Average			297983.3	223533.6	165095.9	1283712.2	10.0	5.9	11.6	16.8

Supplementary Table 2: Summary of top target gene(s) per AF locus

VR#	SNP	Gene	TAD	PCHi-C	LA-PCM	LA	RA	RA fetal	LA fetal	eQTL	Score
1	rs187585530	KIF1B	+	+	++	++	++	+	+	0	13
2	rs880315	KIF1B	+	+	++	++	++	+	+	0	13
3	rs146518726	EPS15	+	+	++	++	++	++	++	0	15
4.1	rs1443926	*HBXIP*	-	-	++	++	++	++	++	0	10
4.2	rs1443926	*ATP5F1*	-	-	++	++	++	++	++	0	10
5	rs4484922	CASQ2	+	-	++	++	++	++	++	0	12
6	rs79187193	PRKAB2	+	+	++	++	++	+	+	0	13
7.1	rs11264280	ADAR	+	+	++	++	++	++	++	0	15
7.2	rs11264280	ADAM15	+	+	++	+	-	+	++	1	15
8	rs72700114	KIFAP3	+	+	+	++	++	++	++	1	18
9	rs503706	KIFAP3	+	+	+	++	++	++	++	1	18
10.1	rs4590732	FMOD	-	+	++	+	++	++	++	0	12
10.2	rs4590732	BTG2	-	+	++	++	++	++	+	0	12
11	rs4951261	NUCKS1	+	-	++	++	++	++	++	1	16
12	rs7919685	JMJD1C	+	+	+	++	++	++	++	0	14
13	rs6480708	SYNPO2L	+	+	++	++	++	++	++	2	23
14.1	rs11001667	*VDAC2*	-	-	++	++	++	++	++	0	10
14.2	rs11001667	*RPS24*	-	-	++	++	++	++	++	0	10
15	rs1044258	MGEA5	+	+	++	++	++	++	++	0	15
16	rs11598047	SLK	+	+	++	++	++	++	++	0	15
17.1	rs1822273	*LDHA*	-	-	++	++	++	++	++	0	10
17.2	rs1822273	*CSRP3*	-	-	++	++	++	++	++	0	10
18.1	rs949078	*ARHGEF12*	-	-	++	++	++	++	++	0	10
18.2	rs949078	*MIR125B1*	+	-	++	++	++	+	+	0	10
18.3	rs949078	*HSPA8*	-	-	++	++	++	++	++	0	10
19	rs76097649	KCNJ5	+	-	+	-	-	+	+	3	17
20	rs10842383	SSPN	+	-	-	++	++	++	++	1	14
21	rs113819537	SSPN	+	-	-	++	++	++	++	1	14
22	rs12809354	PKP2	+	+	++	++	++	++	++	0	15
23.1	rs7978685	ATP5B	+	+	++	++	++	++	++	0	15
23.2	rs7978685	NACA	+	+	++	++	++	++	++	0	15
24	rs35349325	CCT2	+	+	++	++	++	++	++	0	15
25.1	rs11180703	*ATXN7L3B*	-	-	++	++	++	++	++	0	10
25.2	rs11180703	*NAP1L1*	-	-	++	++	++	++	++	0	10
26	rs883079	TBX5	+	+	++	++	++	++	++	2	23
27	rs12810346	TBX5	-	+	++	++	++	++	++	2	21
28	rs12298484	ZNF664	+	+	+	++	++	++	++	1	18
29.1	rs9580438	*SAP18*	-	-	++	++	++	++	++	0	10
29.2	rs9580438	*FGF9*	+	+	++	++	+	-	-	0	10
30.1	rs28631169	MYH6	+	+	++	++	++	++	++	0	15
30.2	rs28631169	PABPN1	+	+	++	++	++	++	++	0	15
31	rs2145587	ARHGAP5	+	+	++	++	++	++	++	0	15
32	rs73241997	SRP54	+	+	+	++	++	++	++	0	14
33	rs2738413	SYNE2	+	+	++	++	++	++	++	2	23
34.1	rs10873299	IRF2BPL	+	+	++	+	+	+	++	0	12
34.2	rs10873299	KIAA1737	+	-	++	++	++	++	++	0	12
35	rs62011291	FAM96A	+	+	+	++	++	++	++	0	14

36	rs12591736	RPLP1	+	-	++	++	++	++	++	0	12
37	rs7172038	NPTN	+	+	++	++	++	++	++	0	15
38	rs12908004	*PSMA4*	-	-	*++*	*++*	*++*	*++*	*++*	*0*	*10*
39	rs12908437	IGF1R	+	+	+	+	+	+	+	1	14
40	rs2286466	NDUFB10	+	+	++	++	++	++	++	0	15
41	rs2359171	DHX38	+	+	++	+	+	+	++	0	12
42.1	rs8073937	GPS2	+	+	++	++	++	++	++	0	15
42.2	rs8073937	EIF4A1	+	+	++	++	++	++	++	0	15
42.3	rs8073937	TRAPPC1	+	+	++	++	++	++	++	0	15
43	rs72811294	MYOCD	+	-	++	++	++	++	++	0	12
44	rs242557	KPNB1	-	+	++	++	++	++	++	0	13
45.1	rs7219869	*PRKAR1A*	-	-	*++*	*++*	*++*	*++*	*++*	*0*	*10*
45.2	rs7219869	*ABCA8*	-	-	*++*	*++*	*++*	*++*	*++*	*0*	*10*
46	rs9953366	C18orf32	+	+	-	++	++	++	++	0	13
47.1	rs6546620	HADHA	+	-	++	++	++	++	++	0	12
47.2	rs6546620	HADHB	+	-	++	++	++	++	++	0	12
48	rs6742276	USP34	+	+	++	++	++	++	++	0	15
49	rs2723064	CEP68	+	+	++	++	+	+	+	2	20
50.1	rs6546553	SNRNP27	+	+	++	+	+	+	+	2	19
50.2	rs6546553	ANXA4	+	+	-	++	++	+	+	2	19
51.1	rs72926475	IMMT	+	+	++	++	++	++	++	0	15
51.2	rs72926475	PTCD3	+	+	++	++	++	++	++	0	15
51.3	rs72926475	KDM3A	+	+	++	++	++	++	++	0	15
52	rs12992412	*MMADHC*	-	-	*+*	*++*	*++*	*++*	*++*	*0*	*9*
53.1	rs56181519	*CIR1*	*+*	-	*+*	*++*	*++*	*++*	*+*	*0*	*10*
53.2	rs56181519	*SP3*	-	-	*++*	*++*	*++*	*++*	*++*	*0*	*10*
53.3	rs56181519	*SCRN3*	*+*	-	*++*	*++*	*++*	*+*	*+*	*0*	*10*
53.4	rs56181519	*WIPF1*	*+*	-	-	*++*	*++*	-	-	*1*	*10*
53.5	rs56181519	*ATP5G3*	-	-	*++*	*++*	*++*	*++*	*++*	*0*	*10*
54	rs2288327	TTN	+	-	++	++	++	++	++	1	16
55	rs295114	SPATS2L	+	+	+	++	++	+	+	0	12
56	rs2145274	*TMX4*	-	-	*++*	*++*	*++*	*++*	*++*	*0*	*10*
57	rs7269123	RPS21	+	+	++	++	++	++	++	0	15
58.1	rs2834618	*TMEM50B*	-	-	*++*	*++*	*++*	*++*	*++*	*0*	*10*
58.2	rs2834618	*ATP5O*	-	-	*++*	*++*	*++*	*++*	*++*	*0*	*10*
59	rs465276	MICAL3	+	+	++	+	+	++	++	0	13
60.1	rs7650482	MKRN2	+	+	++	++	++	+	++	1	18
60.2	rs7650482	CAND2	+	+	+	+	+	+	+	2	18
61	rs73032363	RPL15	+	-	++	++	++	++	++	0	12
62	rs6790396	SCN5A	+	+	++	++	++	++	++	1	19
63	rs2306272	LRIG1	+	-	+	++	++	++	++	0	11
64.1	rs17490701	PHLDB2	+	+	+	++	++	+	+	0	12
64.2	rs17490701	ABHD10	+	+	+	++	++	+	+	0	12
65	rs4855075	MFN1	+	+	++	++	++	++	++	0	15
66	rs9872035	NCBP2	-	+	++	++	++	++	++	0	13
67	rs3822259	ZNF518B	+	+	++	++	++	+	+	0	13
68	rs3960788	UBE2D3	+	+	++	++	++	++	++	1	19
69	rs6847935	PITX2	+	+	+	+	-	-	++	2	17
70	rs55754224	CAMK2D	+	+	+	++	++	++	++	0	14
71	rs10213171	ARHGAP10	+	+	+	++	++	++	++	0	14

72	rs10520260	HAND2	+	+	++	++	++	++	++	0	15
73	rs716845	YTHDC2	+	-	++	++	++	+	+	0	10
74	rs10479177	FAM13B	+	+	+	++	++	+	++	2	21
75	rs174048	NR3C1	+	-	+	++	++	++	++	0	11
76	rs6882776	ATP6V0E1	+	+	+	++	++	++	++	0	14
77	rs73366713	DEK	-	-	+	++	++	++	++	1	13
78	rs34969716	DEK	+	-	+	++	++	++	++	1	15
79.1	rs1307274	*BRD2*	-	-	++	++	++	++	++	*0*	*10*
79.2	rs1307274	*RPS18*	-	-	++	++	++	++	++	*0*	*10*
79.3	rs1307274	*MNF1*	-	-	++	++	++	++	++	*0*	*10*
79.4	rs1307274	*RPL10A*	-	-	++	++	++	++	++	*0*	*10*
80	rs3176326	SRSF3	+	+	++	++	++	++	++	0	15
81	rs6907805	ZNF292	+	+	++	++	++	++	++	0	15
82	rs210632	PLN	-	-	++	++	++	++	++	1	14
83	rs9481842	PLN	+	-	++	++	++	++	++	1	16
84	rs868155	GJA1	+	+	++	++	++	++	++	1	19
85	rs12208899	RPS12	+	+	++	++	++	++	++	0	15
86	rs117984853	PPIL4	+	+	+	++	++	++	+	0	13
87	rs11768850	PDGFA	+	+	+	++	++	+	+	0	12
88	rs55734480	ETV1	+	+	+	++	++	++	++	0	14
89	rs6462078	*TAX1BP1*	-	-	+	++	++	++	++	*0*	*9*
90.1	rs74910854	*BAZ1B*	-	-	++	++	++	++	++	*0*	*10*
90.2	rs74910854	*MDH2*	-	-	++	++	++	++	++	*0*	*10*
90.3	rs74910854	*HSPB1*	-	-	++	++	++	++	++	*0*	*10*
90.4	rs74910854	*YWHAG*	-	-	++	++	++	++	++	*0*	*10*
91.1	rs62483627	HBP1	+	+	++	++	++	++	++	0	15
91.2	rs62483627	DLD	+	+	++	++	++	++	++	0	15
92	rs11773845	CAV1	+	+	++	++	++	++	++	2	23
93.1	rs55985730	CALU	+	+	++	++	++	++	++	0	15
93.2	rs55985730	FLNC	+	+	++	++	++	++	++	0	15
94	rs7789146	KCNH2	+	+	++	+	++	++	+	0	13
95.1	rs7508	PCM1	+	-	++	++	++	++	++	2	20
95.2	rs7508	ASAH1	+	-	++	++	++	++	++	2	20
96	rs7846485	XPO7	+	+	+	++	++	++	++	0	14
97	rs62521286	MTSS1	+	+	+	++	++	+	+	1	16
98	rs35006907	MTSS1	+	+	+	++	++	+	+	1	16
99.1	rs7460121	*ST3GAL1*	-	-	++	++	++	+	+	*0*	*8*
99.2	rs7460121	*MIR30B*	+	-	++	++	++	-	-	*0*	*8*
100	rs6993266	PTK2	+	+	+	++	++	++	++	0	14
101.1	rs4977397	*RRAGA*	-	-	++	++	++	++	++	*0*	*10*
101.2	rs4977397	*RPS6*	-	-	++	++	++	++	++	*0*	*10*
102.1	rs7026071	*FAM120A*	-	-	+	++	++	++	++	*0*	*9*
102.2	rs7026071	*FANCC*	+	-	-	+	+	-	+	*1*	*9*
103	rs4743034	RAD23B	+	-	+	++	++	++	++	0	11
104.1	rs10760361	*RC3H2*	-	-	++	++	++	++	++	*0*	*10*
104.2	rs10760361	*PSMB7*	+	-	+	++	+	++	++	*0*	*10*
104.3	rs10760361	*RPL35*	-	-	++	++	++	++	++	*0*	*10*
104.4	rs10760361	*HSPA5*	-	-	++	++	++	++	++	*0*	*10*

Supplementary Table 3: ATAC-seq motif enrichment

MOTIF	ATAC (55k)	background (100k)	enrichment	Z-statistic	p-value	%ATAC	%bg
HEY2	994	121	8.21	79.4510	<0.0001	1.81	0.22
MEF2A	1482	199	7.45	91.1140	<0.0001	2.69	0.36
CTCF	3325	807	4.12	140.7920	<0.0001	6.05	1.47
Tbx3_custom	13045	3515	3.71	166.1	<0.0001	23.72	6.39
COUP-TF2	462	153	3.02	25.0160	<0.0001	0.84	0.28
MEF2D	1202	549	2.19	28.0100	<0.0001	2.19	1.00
MEF2B	1147	524	2.19	27.3460	<0.0001	2.09	0.95
MEF2C	1825	1033	1.77	24.8770	<0.0001	3.32	1.88
GATA6	735	427	1.72	14.9630	<0.0001	1.34	0.78
T	185	108	1.71	7.4170	<0.0001	0.34	0.20
Gata4	401	244	1.64	10.0730	<0.0001	0.73	0.44
TBX2	252	159	1.58	7.3860	<0.0001	0.46	0.29
Tcf12	670	439	1.53	11.0690	<0.0001	1.22	0.80
HEY1	179	121	1.48	5.2790	<0.0001	0.33	0.22
TBX20	280	217	1.29	4.2850	<0.0001	0.51	0.39
TBX19	189	164	1.15	1.9550	0.0506	0.34	0.30
TBX21	238	225	1.06	0.8680	0.3851	0.43	0.41
Gata1	83467	79732	1.05	NA	NA	151.76	144.97
TBX1/4/5/15	8825	8798	1.00	0.3140	0.7535	16.05	16.00
MEIS1	11482	12131	0.95	6.6740	<0.0001	20.88	22.06
PITX3	596	634	0.94	1.5180	0.1290	1.08	1.15
SMAD2/3/4	598	719	0.83	4.5420	<0.0001	1.09	1.31
Smad4	2319	2796	0.83	9.2590	<0.0001	4.22	5.08
Gata4_custom	11806	14490	0.81	26.0	<0.0001	21.47	26.35
GATA3	33102	46351	0.71	155.1860	<0.0001	60.19	84.27
TCF4	208	296	0.70	5.1290	<0.0001	0.38	0.54
Hand1Tcf3	175	296	0.59	7.0520	<0.0001	0.32	0.54
Nkx2-5	8823	19339	0.46	93.9200	<0.0001	16.04	35.16
Tcf3	88	261	0.34	10.7340	<0.0001	0.16	0.47
Shox2	1476	5466	0.27	56.8680	<0.0001	2.68	9.94
SMAD3	34	132	0.26	8.5400	<0.0001	0.06	0.24

https://www.medcalc.org/calc/test_one_proportion.php

Supplementary Table 4: EMERGE datasets

Dataset	Type	Genome	Tissue	Stage	Source	Deposit	# of Peaks
Conservation	PhastCons	hg19	all	all	Siepel lab	UCSC	10121233 (174584)*
CTCF	ChIP-seq	hg19	HCM cells	cell line	ENCODE	GSE30263	41966
DHSs	Dnase1 hypersensitivity	hg19	cerebellum	adult	ENCODE	GSE32970	104575
DHSs	Dnase1 hypersensitivity	hg19	cerebrum	adult	ENCODE	GSE32970	135930
DHSs	Dnase1 hypersensitivity	hg19	frontal_cortex	adult	ENCODE	GSE32970	110989
DHSs	Dnase1 hypersensitivity	hg19	HCM cells	cell line	ENCODE	GSE26328	317895
DHSs	Dnase1 hypersensitivity	hg19	heart	adult	ENCODE	GSE32970	124949
H3K27ac	ChIP-seq	hg19	brain	fetal 12w post-gest.	Guo lab	GSE63634	77355
H3K27ac	ChIP-seq	hg19	cerebellum	adult	Creyghton lab	GSE40465	174090
H3K27ac	ChIP-seq	hg19	fibroblast cell line	induced myocytes D6	Cao lab	GSE78096	33954
H3K27ac	ChIP-seq	hg19	fibroblast cell line	induced myocytes D11	Cao lab	GSE78096	36649
H3K27ac	ChIP-seq	hg19	heart	fetal 12w post-gest.	Guo lab	GSE63634	49185
H3K27ac	ChIP-seq	hg19	iPSC	induced myocytes	Sakabe lab	GSE77267	19528
H3K27ac	ChIP-seq	hg19	left ventricle	adult	Ren lab	GSE16256	59070
H3K27ac	ChIP-seq	hg19	limbs	gest. E33	Cotney lab	GSE42413	72841
H3K27ac	ChIP-seq	hg19	limbs	gest. E41	Cotney lab	GSE42413	42284
H3K27ac	ChIP-seq	hg19	limbs	gest. E44	Cotney lab	GSE42413	69409
H3K27ac	ChIP-seq	hg19	limbs	gest. E47	Cotney lab	GSE42413	54981
H3K27ac	ChIP-seq	hg19	liver	fetal 12w post-gest.	Guo lab	GSE63634	27741
H3K27ac	ChIP-seq	hg19	right ventricle	adult	Ren lab	GSE16256	126802
H3K27me3	ChIP-seq	hg19	brain	fetal 12w post-gest.	Guo lab	GSE63634	50180
H3K27me3	ChIP-seq	hg19	fibroblast cell line	induced myocytes D6	Cao lab	GSE78096	275
H3K27me3	ChIP-seq	hg19	fibroblast cell line	induced myocytes D11	Cao lab	GSE78096	173
H3K27me3	ChIP-seq	hg19	heart	fetal 12w post-gest.	Guo lab	GSE63634	34795
H3K27me3	ChIP-seq	hg19	hESC	induced myocytes 14d	Murry lab	SRR577565	86945
H3K27me3	ChIP-seq	hg19	iPSC	induced myocytes	Sakabe lab	GSE77267	8952
H3K27me3	ChIP-seq	hg19	liver	fetal 12w post-gest.	Guo lab	GSE63634	41712
H3K36me3	ChIP-seq	hg19	hESC	induced myocytes 14d	Murry lab	SRR577574	118126
H3K4me1	ChIP-seq	hg19	brain	fetal 12w post-gest.	Guo lab	GSE63634	58426
H3K4me1	ChIP-seq	hg19	heart	fetal (d105)	BROAD institute	GSE17312	113536
H3K4me1	ChIP-seq	hg19	heart	fetal 12w post-gest.	Guo lab	GSE63634	59026
H3K4me1	ChIP-seq	hg19	iPSC	induced myocytes	Sakabe lab	GSE77267	13902
H3K4me1	ChIP-seq	hg19	liver	fetal 12w post-gest.	Guo lab	GSE63634	43426
H3K4me3	ChIP-seq	hg19	brain	fetal 12w post-gest.	Guo lab	GSE63634	16745
H3K4me3	ChIP-seq	hg19	fibroblast cell line	induced myocytes D6	Cao lab	GSE78096	18720
H3K4me3	ChIP-seq	hg19	fibroblast cell line	induced myocytes D11	Cao lab	GSE78096	19871
H3K4me3	ChIP-seq	hg19	HCM cells	cell line	ENCODE	GSE35583	49247
H3K4me3	ChIP-seq	hg19	heart	fetal (d105)	BROAD institute	GSE17312	1238
H3K4me3	ChIP-seq	hg19	heart	fetal 12w post-gest.	Guo lab	GSE63634	86937
H3K4me3	ChIP-seq	hg19	hESC	induced myocytes 14d	Murry lab	SRR577568	26960
H3K4me3	ChIP-seq	hg19	iPSC	induced myocytes	Sakabe lab	GSE77267	10760
H3K4me3	ChIP-seq	hg19	liver	fetal 12w post-gest.	Guo lab	GSE63634	11799
H3K9ac	ChIP-seq	hg19	heart	fetal (d105)	BROAD institute	GSE17312	49876
p300	ChIP-seq	hg19	cortex	fetal (gest. week 20)	Pennacchio lab	GSE42881	642876
p300	ChIP-seq	hg19	heart	adult	Pennacchio lab	GSE32587	139824
p300	ChIP-seq	hg19	heart	fetal (gest. week 16)	Pennacchio lab	GSE32587	108384
Pol2	ChIP-seq	hg19	heart	fetal (gest. week 16)	Pennacchio lab	GSE32587	223086
Pol2	ChIP-seq	hg19	iPSC	induced myocytes	Sakabe lab	GSE77267	11332
ATAC	ATAC-seq	hg19	SANLPCs	induced SAN myocytes	Protze lab	unpublished	45389
ATAC	ATAC-seq	hg19	hESC	0d H1 differentiated myocytes	Snyder lab	GSE85330	54378
ATAC	ATAC-seq	hg19	hESC	2d H1 differentiated myocytes	Snyder lab	GSE85330	21979
ATAC	ATAC-seq	hg19	hESC	4d H1 differentiated myocytes	Snyder lab	GSE85330	28991
ATAC	ATAC-seq	hg19	hESC	30d H1 differentiated myocytes	Snyder lab	GSE85330	11931
ATAC	ATAC-seq	hg19	hESC	0d H9 differentiated myocytes	Snyder lab	GSE85330	28417
ATAC	ATAC-seq	hg19	hESC	2d H9 differentiated myocytes	Snyder lab	GSE85330	10980
ATAC	ATAC-seq	hg19	hESC	4d H9 differentiated myocytes	Snyder lab	GSE85330	26580
ATAC	ATAC-seq	hg19	hESC	30d H9 differentiated myocytes	Snyder lab	GSE85330	24661
ATAC	ATAC-seq	hg19	iPSC	0d C15 induced myocytes	Snyder lab	GSE85330	37100
ATAC	ATAC-seq	hg19	iPSC	2d C15 induced myocytes	Snyder lab	GSE85330	35192
ATAC	ATAC-seq	hg19	iPSC	4d C15 induced myocytes	Snyder lab	GSE85330	13920
ATAC	ATAC-seq	hg19	iPSC	30d C15 induced myocytes	Snyder lab	GSE85330	16191
ATAC	ATAC-seq	hg19	iPSC	0d C20 induced myocytes	Snyder lab	GSE85330	27217
ATAC	ATAC-seq	hg19	iPSC	2d C20 induced myocytes	Snyder lab	GSE85330	22065
ATAC	ATAC-seq	hg19	iPSC	4d C20 induced myocytes	Snyder lab	GSE85330	51994
ATAC	ATAC-seq	hg19	iPSC	30d C20 induced myocytes	Snyder lab	GSE85330	19602
ATAC	ATAC-seq	hg19	hESC	cardiac mesoderm H7	Weissman lab	GSE85066	180380
ATAC	ATAC-seq	hg19	left ventricle	induced LV myocytes	Protze lab	unpublished	38335
ATAC	ATAC-seq	hg19	left atrium	adult	Martin lab	unpublished	55045
DHSs	Dnase1 hypersensitivity	hg19	limbs	fetal	ENCODE	GSE90405	95652
Med1	ChIP-seq	hg19	iPSC	induced myocytes 32d	Srivastava lab	GSE85631	15400

*number of ultra-conserved phastCons (score≥600)

Human functional genomic datasets included in merge as possible predictors

Supplementary Table 5: Guide and (q)PCR primer sequences

qPCR primers	Sequence 5' to 3'
Cx43_F	AGTACCCAACAGCAGCAGAC
Cx43_R	AAAATGAAGAGCACCGACAG
Hsf2 F	GTCCAGCTAAACGGCTCCTC
Hsf2 R	ACTGTCTTCCTGAGAGCATAGC
Serinc1_F	TTGCTCGTCGGAGTATGTGT
Serinc1_R	AGCTTTGTAGCCAACCAGGAT
Txnl4b_F	GCAAGAAAGAAGTAGACCAGGC
Txnl4b_R	ACTCAGGTCGGCTGAAGTTT
Dhx38_F	TGACCACCAAGGAGTACATGC
Dhx38_R	CTTCTTTAGCCCGACGACGA
Zfhx3_F	AAGCCGTTGAAGATGCCGAA
Zfhx3_R	TTGCAGGGGAATCCGTCAAC
Hprt_Fw	TGTTGGATATGCCCTTGACT
Hprt_Rv	GATTCAACTTGCGCTCATCT
cTnI F	TCGGGTGGACAAAGTGGATG
cTnI R	AAACTTGCCACGGAGGTCAT
Ube2q1_F	AGGAGCAGACTTCATCCTACTTAAC
Ube2q1_R	CCACCCAGAACATACCCTCC
Adar_F	AAAATCCCAAGCAAGGCAAGC
Adar_R	ATGGTACGGAGTCTTTCCCCA
kcnn3_F	GACCGAACTGTCTTGGGGTT
kcnn3_R	ACTTCCCTTGTGTGATAGGCG
Pmvk_F	CGGAAGATTGTGGAAGGCGT
Pmvk_R	GGATGTCAGATGTCCTCCGTG
Pbxip1_F	AACTTACCCTCCAGTCCCAGA
Pbxip1_R	GGTCCTCCACATCTACGGGT
Pygo2_F	AGCCCTGAAAAGAAGCGAAGA
Pygo2_R	TTCAAGGGCTTCCCACCATC
Shc1_F	GAATCCACCGAAGCTGGTCA
Shc1_R	CGAAGCCTCATATCTACCACCC
Flad1_F	CCATTTCAGTCCCACCGACC
Flad1_R	GGCTCAGGCACTTCAGGG
Zbtb7b_F	AGAAGCCCTTTGCCTGTGAG
Zbtb7b_R	TGTGGTTCTTGAGGTCGTAGC
Adam15_F	CACAGCAGATGACAGGCACTA
Adam15_R	GGATTGGATCGGTCAGCCAG
Efna1_F	GGCAAGGAGTTCAAGGAAGGA
Efna1_R	TGACATGGGCCTGGGGATTA
Dpm3_F	CGGAGCCAACATGACGAAGT
Dpm3_R	CAGCGGAGACCAACAGGTAG
Slc50a1_F	GCTCCGCCTCTTGGTCTATT
Slc50a1_R	CGGTACTTCCTGTCTTGCTCT
Krtcap2_F	TTCTCCTCTGCTTGCTGTTGG
Krtcap2_R	CAGTGATTTTGGCCGGTGTG
Rpl32_F	GGTGAAGCCCAAGATCGTCA
Rpl32_R	TCTGGCCCTTGAACCTTCTC
ppia_F	GGGTGGTGACTTTACACGCC
ppia_R	CTTGCCATCCAGCCATTCAG
rpl4_F	GCCGCTGGTGGTTGAAGATAA
rpl4_R	CGTCGGTTTCTCATTTTGCCC

CRISPR guides	Sequence 5' to 3'
Gja1 guide 1	CTCTACGATAGGCATAATAATGG
Gja1 guide 2	GAGTGCCAATATCAGATTCCTGG
Kcnn3 guide1	GGCCTGGACTCTGTTCACCT
Kcnn3 guide2	GGTGGTTCATCCCCATTGCA
Zfhx3 guide 1	TAGGCAGAGCTGTCCTAATAAC
Zfhx3 guide 2	TAGGACTCCGCTATGACAGCAG

Luciferase assay	Sequence 5' to 3'
rs2177843_F	AGAGTGTTGTTCTCCAAGGCAA
rs2177843_R	AGGAGCATGGTACCTTGAGTG
rs9481825_F	AGGTGACAACCAATTCCACCTT
rs9481825_R	CTGGCCGAGTAGTCACCTTTT
rs939601_F	TGATTCCTGGATTCTGCCATCT
rs939601_R	GAGCACAGGATCTCTGCTTGA
rs7312625_F	CCTCGGTTGGAATGTTCCCT
rs7312625_R	AGATGTTGGCACTCAAGGCA
rs2270188_F	CGTCAGGTTGGCTGACAGTT
rs2270188_R	CAGGGGCAAAGTTGAAGCAG
rs10786775_F	CATGCCTGTGGTCCCAGTTA
rs10786775_R	AACAACCACATGATGTTCCCTG
rs2595104_F	CCAGCCAATCAAGCAACGAC
rs2595104_R	GCCGTTGAATGTCTCTTCTCCA
rs9302644_F	AGGAATACTGGGGTAAGGAGGT
rs9302644_R	AACACACGAAGGGCTGTTCA
rs2739197_F	CACACCAAATCTCCTGGGACG
rs2739197_R	GGAAACTCCGCGCCTAGAG
rs10821415_F	CAGAGCAGCAGGTGGGTATT
rs10821415_R	GCTGTGCCAAGTACTAAGTGGA
rs2278933_F	ACATCCCACTGGCACTTGAG
rs2278933_R	CCTGTGCTAAGCTAGTGGCA

Supplementary Methods

Nuclei isolation

Nuclear isolation was performed as previously published[1] using an adapted protocol. Samples were kept at 4°C throughout the procedure. In short, approximately 100mg tissue was trimmed to 2mm pieces, and homogenized in lysis buffer containing RNase inhibitor for 10 seconds at maximum speed using an Ultra-Turrax homogenizer. Samples were homogenized further using a douncer with loose pestle (10 strokes). After a 10-minute incubation in the lysis buffer, 10 strokes were performed with a tight pestle. The lysis procedure was monitored by light microscopy to ensure complete tissue and cell lysis and efficient nuclear extraction. The crude lysate was consecutively passed through mesh filters with pore size of 100 µm and 30 µm. The final lysate was spun at 1000x*g* for 5 minutes and the resulting pellet was resuspended in 500 µl staining buffer (5% BSA in PBS) supplemented with RNAse inhibitor.

Library preparation and sequencing

For adult left and right atria, whole transcriptome amplification was performed using NuGEN's Ovation RNA-Seq V2 kit (San Carlos, CA) according to manufacturer's instructions. For fetal left and right atria, Truseq Stranded Total RNA Library Preparation kit (Illumina, San Diego, CA) was used according to manufacturer's instructions (with 15 PCR cycles). For whole tissue, 50 ng of RNA was used as input, for RNA isolated from cardiomyocyte nuclei approximately 600 pg was used as input. 500 ng cDNA was fragmented to 200-400 bp using Covaris S2 Focused ultrasonicator (Woburn, MA) and the fragmentation parameters described in the NuGEN ENCORE NGS library preparation protocol. Library preparation was done with Ovation Ultralow System V2 using 10 pg-100 ng of fragmented double-stranded DNA. Sequencing was performed on an Illumina Hiseq4000 sequencing system (50-bp single reads).

Differential expression analysis

Reads were mapped to hg19 build of the human transcriptome using STAR[2]. Differential expression analysis was performed using the DESeq2 package based on a model using the negative binomial distribution[3]. The false discovery rate (FDR) method of Benjamini-Hochberg ($p<0.05$) was used to correct p-values for multiple testing.

Unsupervised hierarchical clustering was performed on genes differentially expressed using the R package pheatmap, version 1.0.8. (http://cran.r-project.org/web/packages/pheatmap/index.html). DAVID[4] was used to find overrepresented gene ontology (GO) terms and Kegg pathways in the categories 'biological process' and 'molecular function'. Benjamini–Hochberg correction was performed for multiple testing-controlled P values. Significantly enriched terms were functionally grouped and visualized. The highest significant term of the group was displayed as leading term. Datasets were normalized to reference genes expressed in all datasets (*RPL32*, *RPL4* and *H2AF2* [5]), after which expression was set per dataset at the median, and high expression at the third quartile to enable categorization of expression for target gene identification. HOMER analysis was performed as described [6].

EMERGE

Enrichment of putative heart enhancers was tested as predicted by EMERGE. A total of 70 selected functional genomic datasets from a variety of cardiomyocyte (CM) containing tissues, including whole hearts and CMs differentiated from human embryonic and induced pluripotent stem cells (both publicly available and in-house), was integrated using EMERGE. This included ChIP-seq data of enhancer-associated histone methylation marks and transcription factor binding sites and chromatin accessibility data as assessed by DNase1-hypersensitivity and ATAC-seq. Subsequently an overall heart enhancer prediction track was generated by assigning weights to all 70 selected functional genomic datasets through a logistic regression modelling approach that determines a best fit on 84 validated heart enhancers [7].

ATAC-seq footprinting

ATAC-seq footprinting was performed as previously described[8].

qPCR

Total RNA was isolated from using TRIzol Reagent according to the manufacturer's protocol (Thermo Fisher Scientific, Bleiswijk, the Netherlands). Concentration of RNA was measured using Nanodrop. Genomic DNA was removed using DNase treatment. The Superscript II system (Thermo Fisher Scientific) and oligo dT primers (125 µM) were used to generate cDNA, and expression levels of different genes were assessed with qPCR using the LightCycler 2.0 Real-Time PCR system (Roche Life Science, Indianapolis, IN). Primer pairs spanned at least one intron, and they are listed in Suppl. Table 1. The qPCR reaction was performed with LightCycler 480 SYBR Green I Master (Roche), primers (1 µM) and cDNA (equivalent to 5 ng RNA). The amplification protocol consisted of 5 min 95 °C followed by 45 cycles of 10 sec 95°C, 20 sec 60°C and 20 sec 72°C. Data was analyzed using *LinRegPCR*.[9] We used two reference genes per experiment based on literature to normalize gene expression (*Hprt, Ppia, Rpl4* and *Rpl32)*.[5]

Cell culture and Luciferase assay

Primers were designed for selected AF variants to amplify fragments of 400-900 bp (For primers, see Suppl. Table 1) on DNA heterozygous for the selected variant. The pGL2-Promoter Vector (Promega, Madison, WI) was linearized with XcmI (New England Biolabs, Ipswich, MA) to create a T-overhang and agarose-gel isolated. PCR products containing both alleles of the selected variants were ligated using TA-cloning to the pGL2-Promoter Vector. Recombinant plasmid DNA was purified with the PureLink Quick Plasmid Miniprep kit (Thermo Fisher Scientific). Plasmids that were identical except for the different alleles of the variant were selected using Sanger sequencing.

iAM-1 cells were plated at a cell density of 4×10^4 cells per cm^2 and cultured as described [10]. At day 0 of cardiomyogenic differentiation, 500 ng pGL2-Promoter Vector DNA as well as 25 ng pRL-TK Renilla vector

(Promega) as normalization control was transfected into the cells *in triplo* in a 24-well plate using Lipofectamine 3000 (Thermo Fisher Scientific). At day 9 of cardiomyogenic differentiation the cells were harvested. Luciferase measurements were performed on a Promega Turner Biosystems Modulus Multimode Reader luminometer.

Supplementary Note 1

Description of Supplementary Datasets and programs (Galaxy, Excel) used to produce results in manuscript *"Identification of atrial fibrillation associated genes and functional non-coding variants"* **.**

**Supplementary data file**	**title**	**publication**
8	lead SNPs	Roselli, C. *et al.* Multi-ethnic genome-wide association study for atrial fibrillation. (2018).
9	Hi-C data (TADs)	http://promoter.bx.psu.edu/hi-c/view.php
10	Variant regions: AF GWAS SNPs $p<10^{-6}$	Roselli, C. *et al.* Multi-ethnic genome-wide association study for atrial fibrillation. (2018).
11	AF GWAS SNPs $p<10^{-4}$	Roselli, C. *et al.* Multi-ethnic genome-wide association study for atrial fibrillation. (2018).
12	SNPs p<0.05 within variant regions	Subthreshold SNPs (Supplementary Data file 11) within varaint regions (cooridinates of Supplementary Data file 10)
13	Genome-wide PCHi-C	Montefiori, L. E. *et al.* A promoter interaction map for cardiovascular disease genetics. *bioRxiv* 340869 (2018). Doi:10.1101/340869
14	Promoters contacted by the variant regions	Joining of Supplementary Data file 10 "Variant regions: AF GWAS SNPs $p<10^{-6}$" and Supplementary Data file 13
2	Human adult and fetal left and right atrial RNA-seqs	GSE127856 https://www.ncbi.nlm.nih.gov/geo/query/acc.cgi?acc=GSE127856 accessible with token: olifwwcorbqbxch
14	Normalized RNA-seq and PCHi-C	Joining of Supplementary Data file 7 and Suppl Table 2. Normalized to *RPL32*, *RPL4* and *H2AF2.*
15	Genome-wide ATAC-seq peaks	Unpublished data Zhang, and Hill et al., 2019
4	Genome-wide EMERGE peaks	Van Duijvenboden, K., De Boer, B. A., Capon, N., Ruijter, J. M. & Christoffels, V. M. EMERGE: A flexible modelling framework to predict genomic regulatory elements from genomic signatures. *Nucleic Acids Res.* **44,** (2015). New training of EMERGE in this manuscript, see Supplementary Table 3 for list of newly incorporated datasets.
	**Program used**	
A	Galaxy	https://usegalaxy.org/ used online, free subscription
A1	general text tools → join two datasets side by side	Used to merge 2 datasets on a specific field (e.g. to extract only the selected genes of interest from RNA-seq datasets, and get these values into the same Supplementary Data file with e.g. TAD or PCHi-C Supplementary Data file)
A2	Common genomic tools → operate on genomic intervals → join	Used to extract e.g. all SNPs with $p<10^{-4}$ from a Supplementary Data file based on their coordinates falling within the coordinates of a variant region.

	the intervals of two datasets side-by-side	
A3	general text tools → group	Used to perform count and sum operations on datasets. E.g. to count the number of times a certain interaction with a specific gene was found in PCHi-C dataset, as well as the added value of interaction strength (interaction of the variant region with gene X was found 12 times with a cumulative interaction strength of 20.5)

Target gene score calculation:

- Using Supplementary Data file 8 "lead SNPs", we selected the Topologically Associated Domains (TADs) that these variants were in (using http://promoter.bx.psu.edu/hi-c/view.php) ; resulting in Supplementary Data file 9 "Hi-C data (TADs)" containing coordinates of relevant TADs.
- Using Supplementary Data file 8 "lead SNPs", we determined the potential functional range of 1 Mb up- and downstream of these lead SNPs by simply adding and subtracting 1 Mb to the SNP coordinate (taking into account start and end of the chromosome).
- Using Supplementary Data file 10 "Variant regions: AF GWAS SNPs $p<10^{-6}$" we determined the variant regions, defined as the first to last variant with $p<10^{-6}$ in LD with the lead SNPs. To extract the subthreshold variants per variant region, we used function A2 of Galaxy to find only the "AF GWAS SNPs $p<10^{-4}$" out of Supplementary Data file 11 that lie within the variant regions. Resulting in Supplementary Data file 12 "SNPs $p<10^{-4}$ within variant regions"
- Using coordinates of Supplementary Data file 10 "Variant regions: AF GWAS SNPs $p<10^{-6}$" and Supplementary Data file 13 "Genome-wide PCHi-C", we extracted only the promoters of genes contacted by the variant regions (using Galaxy function A2). This Supplementary Data file we summarized using Galaxy function A3 to indicate per gene in contact with the variant region 1) the added interaction strength and 2) the number of interactions with that promoter (Variant region PCHi-C interactions).
- Using coordinates of 1 Mb up- and downstream of lead SNP Supplementary Data file 8, we extracted all potential target genes from Supplementary Data file 2 "Human adult and fetal left and right atrial RNA-seqs".
- To be able to compare between datasets, we merged Supplementary Data file 2 "Human adult and fetal left and right atrial RNA-seqs" and "Variant region PCHi-C interactions" and normalized expression using reference genes *RPL32*, *RPL4* and *H2AF2.* This culminated in Supplementary Data file 14 "Normalized RNA-seq and PCHi-C". Using this file we converted the tags to a categorized score of "No expression (0)", "Expression (1)" and "High expression (2)" based on normalized expression (Supplementary Figure 4) (using excel):

**Dataset**	**Low Expression (0)**	**Expression (1)**	**High expression (2)**
RNA-seq LA-PCM	0<>10 tags	10<>40 tags	>40 tags
RNA-seq LA	0<>4 tags	4<>20 tags	>20 tags
RNA-seq RA	0<>3 tags	3<>20 tags	>20 tags

RNA-seq RA fetal	0<>3 tags	3<>8 tags	>8 tags
RNA-seq LA fetal	0<>3 tags	3<>8 tags	>8 tags
PCHi-C	0<>0 tags	>0 tags	-

- Using Galaxy function A1 and A2 we merged Supplementary Data file 8 “lead SNPs”, Supplementary Data file 9 “Hi-C data (TADs)”, Supplementary Data file 14 “Normalized RNA-seq and PCHi-C”. Moreover we manually added the genes that have been implicated in eQTLs in excel. This culminated in Supplementary Data file 2 “Target gene scoring table (TAD, PCHi-C, RNA-seqs and eQTL)”.

Identification of cardiac REs and variant REs:

- Using Galaxy function A2 and coordinates of variant regions from Supplementary Data file 10 “Variant regions: AF GWAS SNPs $p<10^{-6}$” we extracted ATAC-seq peaks from genome-wide ATAC-seq peak calling resulting in Supplementary Data file 15 “Genome-wide ATAC-seq peaks” that lie within the variant regions Supplementary Data file 3.
- Using Galaxy function A2 and coordinates of variant regions of Supplementary Data file 10 “Variant regions: AF GWAS SNPs $p<10^{-6}$” we extracted EMERGE peaks from Supplementary Data file “Genome-wide EMERGE peaks” that lie within the variant regions resulting in Supplementary Data file 4.
- Again using Galaxy function A2, we merged Supplementary Data file 12 “SNPs $p<10^{-4}$ within variant regions” with Supplementary Data file 15 “Genome-wide ATAC-seq peaks” and Genome-wide EMERGE peaks to get Supplementary Data file 5 “Variants within ATAC-seq or EMERGE peak calling”. These are the potential regulatory potential altering variants.
- To obtain the variants that are potentially altering expression of specific AF target genes, we merged the following files (using Galaxy function A1 and A2): Supplementary Data file 12 “SNPs $p<10^{-4}$ within variant regions”, and Supplementary Data file 14 “Promoters contacted by the variant regions”, Supplementary Data file 5 “Variants within ATAC-seq or EMERGE peak calling” and Supplementary Data file 1 “Target gene scoring table (TAD, PCHi-C, RNA-seqs and eQTL)”. This resulted in Supplementary Data file 6 “Potential variant enhancers and their target genes”. In this Supplementary Data file all SNPs that lie in potential regulatory regions (ATAC+EMERGE) and their potential target genes (PCHi-C) with AF target gene score (above score 11) are summarized.

Supplementary References

1. Bergmann, O. & Jovinge, S. Isolation of Cardiomyocyte Nuclei from Post-mortem Tissue. *J. Vis. Exp.* 4–9 (2012). doi:10.3791/4205

2. Dobin, A. *et al.* STAR: Ultrafast universal RNA-seq aligner. *Bioinformatics* (2013). doi:10.1093/bioinformatics/bts635

3. Love, M. I., Huber, W. & Anders, S. Moderated estimation of fold change and dispersion for RNA-seq data with DESeq2. *Genome Biol.* (2014). doi:10.1186/s13059-014-0550-8

4. Huang, D. W., Sherman, B. T. & Lempicki, R. A. Systematic and integrative analysis of large gene lists using DAVID bioinformatics resources. *Nat. Protoc.* (2009). doi:10.1038/nprot.2008.211

5. Ruiz-Villalba, A. *et al.* Reference genes for gene expression studies in the mouse heart. *Sci. Rep.* (2017). doi:10.1038/s41598-017-00043-9

6. Heinz, S. *et al.* Simple Combinations of Lineage-Determining Transcription Factors Prime cis-Regulatory Elements Required for Macrophage and B Cell Identities. *Mol. Cell* (2010). doi:10.1016/j.molcel.2010.05.004

7. Visel, A., Minovitsky, S., Dubchak, I. & Pennacchio, L. A. VISTA Enhancer Browser - A database of tissue-specific human enhancers. *Nucleic Acids Res.* **35,** 88–92 (2007).

8. Wei, Z., Zhang, W., Fang, H., Li, Y. & Wang, X. esATAC: an easy-to-use systematic pipeline for ATAC-seq data analysis. *Bioinformatics* (2018). doi:10.1093/bioinformatics/bty141

9. Ruijter, J. M. *et al.* Amplification efficiency: Linking baseline and bias in the analysis of quantitative PCR data. *Nucleic Acids Res.* (2009). doi:10.1093/nar/gkp045

10. Liu, J. *et al.* Generation and primary characterization of iAM-1, a versatile new line of conditionally immortalized atrial myocytes with preserved cardiomyogenic differentiation capacity. *Cardiovasc. Res.* 1–12 (2018). doi:10.1093/cvr/cvy134
