# Supplementary material for: Identification of atrial fibrillation associated genes and functional non-coding variants
Source: Nat Commun. 2019 Oct 18;10:4755. doi: 10.1038/s41467-019-12721-5 (PMC6802215; doi:10.1038/s41467-019-12721-5)
Supplement: Supplementary file 3 — Reporting Summary [file 41467_2019_12721_MOESM3_ESM.pdf]

nature research

Corresponding author(s): Vincent M. Christoffels

Last updated by author(s): 19/09/19

Reporting Summary

Nature Research wishes to improve the reproducibility of the work that we publish. This form provides structure for consistency and transparency in reporting. For further information on Nature Research policies, see Authors & Referees and the Editorial Policy Checklist .

Statistics

For all statistical analyses, confirm that the following items are present in the figure legend, table legend, main text, or Methods section.

n/a	Confirmed	
[ ]	[x]	The exact sample size (*n*) for each experimental group/condition, given as a discrete number and unit of measurement
[ ]	[x]	A statement on whether measurements were taken from distinct samples or whether the same sample was measured repeatedly
[ ]	[x]	The statistical test(s) used AND whether they are one- or two-sided<br>*Only common tests should be described solely by name; describe more complex techniques in the Methods section.*
[x]	[ ]	A description of all covariates tested
[ ]	[x]	A description of any assumptions or corrections, such as tests of normality and adjustment for multiple comparisons
[ ]	[x]	A full description of the statistical parameters including central tendency (e.g. means) or other basic estimates (e.g. regression coefficient) AND variation (e.g. standard deviation) or associated estimates of uncertainty (e.g. confidence intervals)
[ ]	[x]	For null hypothesis testing, the test statistic (e.g. *F*, *t*, *r*) with confidence intervals, effect sizes, degrees of freedom and *P* value noted<br>*Give P values as exact values whenever suitable.*
[x]	[ ]	For Bayesian analysis, information on the choice of priors and Markov chain Monte Carlo settings
[x]	[ ]	For hierarchical and complex designs, identification of the appropriate level for tests and full reporting of outcomes
[x]	[ ]	Estimates of effect sizes (e.g. Cohen's *d*, Pearson's *r*), indicating how they were calculated

Our web collection on statistics for biologists contains articles on many of the points above.

Software and code

Policy information about availability of computer code

Data collection	no software was used for data collection
Data analysis	Galaxy (https://usegalaxy.org/ ;Galaxy version 19.01.rc1) was used for the joining of files based on either genome coordinates or gene name (EMSEMBLE Biomart).<br>EMERGE program published (van Duijvenboden, K. et al. EMERGE: a flexible modelling framework to predict genomic regulatory elements from genomic signatures. Nucleic Acids Res. 2016 Mar 18;44(5)e42. doi: 10.1093/nar/gkv1144).

For manuscripts utilizing custom algorithms or software that are central to the research but not yet described in published literature, software must be made available to editors/reviewers. We strongly encourage code deposition in a community repository (e.g. GitHub). See the Nature Research guidelines for submitting code & software for further information.

Data

Policy information about availability of data

All manuscripts must include a data availability statement. This statement should provide the following information, where applicable:

- Accession codes, unique identifiers, or web links for publicly available datasets
- A list of figures that have associated raw data
- A description of any restrictions on data availability

RNA-seq data that supports the findings of this study have been deposited in NCBI's Gene Expression Omnibus (Edgar et al., 2002) and are accessible through GEO Series accession number GSE127856 (https://www.ncbi.nlm.nih.gov/geo/query/acc.cgi?acc=GSE127856).
To review GEO accession GSE127856:
Go to https://www.ncbi.nlm.nih.gov/geo/query/acc.cgi?acc=GSE127856
Enter token olifwwcorbqbxch into the box.

ATAC-seq data: "Zhang and Hill et al., 2019". Human ATAC sequencing raw sequencing files are available in the NCBI dbGAP database under accession code

phs001539.v1.p1. ATAC sequencing post alignment bed format files are available on the Broad Institute's Cardiovascular Disease Knowledge Portal (broadcvdi.org/informational/data) and for direct download using gsutil from gs://cvdi_epigenome/Human/Left_Atrium/.

PCHi-C data published (Montefiori, L. E. et al. A promoter interaction map for cardiovascular disease genetics. bioRxiv 340869 (2018). doi:10.1101/340869).

EMERGE: The heart enhancer prediction track generated with EMERGE for this study is made available via the Nature Communications file transfer site. Including a list of all the functional genomic datasets that were integrated in EMERGE to generate this prediction.

Field-specific reporting

Please select the one below that is the best fit for your research. If you are not sure, read the appropriate sections before making your selection.

- [x] Life sciences
- [ ] Behavioural & social sciences
- [ ] Ecological, evolutionary & environmental sciences

For a reference copy of the document with all sections, see nature.com/documents/nr-reporting-summary-flat.pdf

Life sciences study design

All studies must disclose on these points even when the disclosure is negative.

Sample size	No sample-size calculation was performed in this study. For initial analysis we started with a group size of n=3 and we continue with an in-dept analysis (i.e. qPCR) by increasing the group size as described in this study to observe differences between groups. For RNA-seq we based our sample size on previous performed studies where a group size of n=3 was sufficient for these analyses.
Data exclusions	No data were excluded from the analyses.
Replication	All attempts at replication for qPCR were successful. RNA-seq was not replicated, because of scarcity of the available tissue.
Randomization	Randomization not relevant to this study. Mice were selected based on genotype.
Blinding	Not relevant to this study.

Reporting for specific materials, systems and methods

We require information from authors about some types of materials, experimental systems and methods used in many studies. Here, indicate whether each material, system or method listed is relevant to your study. If you are not sure if a list item applies to your research, read the appropriate section before selecting a response.

Materials & experimental systems

n/a	Involved in the study	
[ ]	[x]	Antibodies
[ ]	[x]	Eukaryotic cell lines
[x]	[ ]	Palaeontology
[ ]	[x]	Animals and other organisms
[x]	[ ]	Human research participants
[x]	[ ]	Clinical data

Methods

n/a	Involved in the study	
[x]	[ ]	ChIP-seq
[ ]	[x]	Flow cytometry
[x]	[ ]	MRI-based neuroimaging

Antibodies

Antibodies used	For cardiomyocyte-specific nuclear RNA-seq: PCM-1. (Sigma-Aldrich; HPA023370)<br>Alexa Fluor 647-conjugated donkey-anti-rabbit 647 antibodies (ThermoFisher Scientific;A-31573)
Validation	Anti-PCM1 antibody produced in rabbit has been used as a primary antibody in immunofluorescence. It has also been used for immunohistochemistry study and for flow cytometry and magnetic-assisted cell sorting assay.<br>All Prestige Antibodies®Powered by Atlas Antibodies is developed and validated by the Human Protein Atlas (HPA) project (www.proteinatlas.org). Each antibody is tested by immunohistochemistry against hundreds of normal and disease tissues. These images can be viewed on the Human Protein Atlas (HPA) site by clicking on the Image Gallery link.<br><br>Alexa 647: To minimize cross-reactivity, these donkey anti-rabbit IgG whole antibodies have been affinity-purified and show a published cross-reactivity to rat IgG. Cross-adsorption or pre-adsorption is a purification step to increase specificity of the antibody resulting in higher sensitivity and less background staining. The secondary antibody solution is passed through a column matrix containing immobilized serum proteins from potentially cross-reactive species. Only the nonspecific-binding secondary antibodies are captured in the column, and the highly specific secondaries flow through. The benefits of this extra step are

apparent in multiplexing/multicolor-staining experiments (e.g., flow cytometry) where there is potential cross-reactivity with other primary antibodies or in tissue/cell fluorescent staining experiments where there may be the presence of endogenous immunoglobulins. (https://www.thermofisher.com/antibody/product/Donkey-anti-Rabbit-IgG-H-L-Highly-Cross-Adsorbed-Secondary-Antibody-Polyclonal/A-31573)

Eukaryotic cell lines

Policy information about cell lines

Cell line source(s)	iAM-1 cell line was published, cells from this initial batch were used:<br>Liu, J. et al. Generation and primary characterization of iAM-1, a versatile new line of conditionally immortalized atrial myocytes with preserved cardiomyogenic differentiation capacity. Cardiovasc. Res. 1–12 (2018). doi:10.1093/cvr/cvy134
Authentication	New cell line, extensively characterized.
Mycoplasma contamination	All cell lines tested negative for mycoplasma contamination.
Commonly misidentified lines (See ICLAC register)	Not applicable.

Animals and other organisms

Policy information about studies involving animals; ARRIVE guidelines recommended for reporting animal research

Laboratory animals	Mouse; FVB/NRj; sexes: males and females; age: 0 - 3 months; embryonic day: E15.5
Wild animals	The study did not involve wild animals.
Field-collected samples	The study did not involve samples collected from the field.
Ethics oversight	All animal experiments were done under DAE285 (IvD Amsterdam University Centers). Animal care and experiments conform to the Directive 2010/63/EU of the European Parliament. All animal work was approved by the Animal Experimental Committee of the Academic Medical Center, Amsterdam, and was carried out in compliance with Dutch government guidelines.

Note that full information on the approval of the study protocol must also be provided in the manuscript.

Flow Cytometry

Plots

Confirm that:

- [x] The axis labels state the marker and fluorochrome used (e.g. CD4-FITC).
- [x] The axis scales are clearly visible. Include numbers along axes only for bottom left plot of group (a 'group' is an analysis of identical markers).
- [x] All plots are contour plots with outliers or pseudocolor plots.
- [x] A numerical value for number of cells or percentage (with statistics) is provided.

Methodology

Sample preparation	RNA-seq: On human left atrial tissue, nuclear isolation was performed as described before (and in the methods.
Instrument	BD Influx
Software	Influx software
Cell population abundance	1.65% of total events, 10.89% of DAPI-positive nuclei were PCM-1 positive (=Cardiomyocyte nuclei).
Gating strategy	Initial gating was set manually, removing nuclear debri and duplicates. Second gating was set on DAPI-positive fraction (FSC 1,533-4,529). Also based on a negative control (no secondary antibody Alexa Fluor 647), gating for PCM-1 positive fraction was set (FSC 1,637-6,871).

- [x] Tick this box to confirm that a figure exemplifying the gating strategy is provided in the Supplementary Information.
