# Supplementary material for: Identification of atrial fibrillation associated genes and functional non-coding variants
Source: Nat Commun. 2019 Oct 18;10:4755. doi: 10.1038/s41467-019-12721-5 (PMC6802215; doi:10.1038/s41467-019-12721-5)
Supplement: Supplementary file 4 — Description of Additional Supplementary Files [file 41467_2019_12721_MOESM4_ESM.pdf]

Description of Additional Supplementary Files

Supplementary Data 1: Target gene scoring

Supplementary Data 2: Variants within ATAC-seq or EMERGE peaks

Supplementary Data 3: Potential variant enhancers and their target genes

Supplementary Data 4: Potential variant enhancers disturbing TFBM

Supplementary Data 5: lead SNPs

Supplementary Data 6: TADs

Supplementary Data 7: Variant regions

Supplementary Data 8: SNPs p_0.0001

Supplementary Data 9: Subthreshold SNPs in variant regions

Supplementary Data 10: Promoter-capture Hi_C

Supplementary Data 11: RNA_seq

Supplementary Data 12: Normalized RNA_seq and PCHiC data

Supplementary Data 13: ATAC_seq peaks in variant regions

Supplementary Data 14: EMERGE peaks in variant regions

Supplementary Data 15: ATAC-seq left atrium adult genome-wide peaks
